# Supplementary material for: Rare jackpot individuals drive rapid adaptation in Threespine Stickleback
Source: Nat Commun. 2026 Mar 30;17:4614. doi: 10.1038/s41467-026-71236-y (PMC13199453; doi:10.1038/s41467-026-71236-y)
Supplement: Supplementary file 1 — Supplementary Information [file 41467_2026_71236_MOESM1_ESM.pdf]

Rare Jackpot Individuals Drive Rapid Adaptation in Threespine Stickleback

AUTHORS: Alexander Kwakye[1,2], Kerry Reid[3], Matthew A. Wund[4], David C. Heins[5], Michael A. Bell[6], Krishna R. Veeramah[1*]

AFFILIATIONS:

1. Department of Ecology and Evolution, Stony Brook University, Stony Brook, NY 11794, USA
2. The Graduate Program in Genetics, Stony Brook University, Stony Brook, NY 11794, USA
3. Department of Ecology and Evolutionary Biology, Yale University, New Haven, CT 06520, USA
4. Department of Biology, The College of New Jersey, Ewing, NJ 08628, USA
5. Department of Ecology & Evolutionary Biology, Tulane University, New Orleans, LA 70118, USA
6. University of California Museum of Paleontology, University of California, Berkeley, CA 94720, USA

*Corresponding Author:krishna.veeramah@stonybrook.edu

Supplementary notes

Supplementary Note 1: Establishment, temporal sampling and study of rapid adaptation in Scout Lake

In 1990, Threespine Sticklebacks (TS) that resembled anadromous sticklebacks were identified in Loberg Lake (61.559N, 149.262W)[1]. Loberg Lake is a natural kettle lake with a surface area of 4.45 hectares and a mean depth of 5.4 meters in Matanuska-Susitna Borough, Alaska. The lake was previously treated with the broad-spectrum piscicide rotenone to enhance recreational fishing. Threespine Sticklebacks were not detected until 1990. From 1990, Threespine Stickleback samples ranging in size from 59 (1990) to 6500 (1999) were collected from this lake. In 1990, all adults were complete lateral plate morphs, for which anadromous stickleback in Cook Inlet are monomorphic, but some juveniles were low morphs, which are monomorphic in most Cook Inlet lake populations. Thus, the population appeared to be rapidly evolving freshwater phenotypes and annual population sampling was initiated. By 1994, about 45% of the Loberg Lake stickleback sampled were low plate morphs. The frequency of low morphs increased to about 75% in 2001[1]. Other traits have diverged from the condition of anadromous stickleback toward that of freshwater ones[2,3]. This phenotypic change was one of the earliest cases of rapid phenotypic evolution in Threespine Stickleback[2]. A channel at the south end of the lake apparently discharges into Spring Creek, which is in the same drainage as Rabbit Slough, was probably the route by which Loberg Lake was colonized by anadromous stickleback.

Rapid evolution in Loberg Lake and Klepaker's (1993)[4] previous report of rapid evolution in a Norwegian population indicated that oceanic stickleback adapt to freshwater fast enough to study using contemporary time series. To add replicates and greater experimental control, where the source and number of Threespine Stickleback founders are known, we released about 3000 anadromous Threespine Stickleback (TS) from Rabbit Slough (61.536 N, 149.253 W), Matanuska-Susitna Borough, Alaska into each of three lakes, including Scout Lake[5]. Before introducing TS into these lakes, Northern Pike, which feeds on small fish, including TS, had invaded them. The Alaska Department of Fish and Game treated these lakes with rotenone to exterminate the invasive Northern Pike. After rotenone treatment, tests for toxicity using caged fish were performed in the lakes to determine if they could survive. They treated Scout Lake in the fall of 2009, but it was still too toxic to introduce Threespine Stickleback before the stickleback breeding season ended in June and July of 2010. After further toxicity testing and about 66 trap hours on 11 May 2010, no TS or Northern pike were captured[5]. Thus, we released 3047 anadromous sticklebacks from Rabbit Slough into Scout Lake in June and July 2011[5].

Scout Lake (60.5353N, 150.8322W) is on the Kenai Peninsula, Alaska, USA. It is about 75 m above sea level with a maximum depth of 6.1m. The lake's surface area is about 38.5 ha in a sparsely developed suburban area in Sterling. It is a natural seepage lake without any stream outlets. Aside from TS, the lake is stocked with coho salmon (*Oncorhynchus kisutch*), Rainbow Trout (*Oncorhynchus mykiss*), and Arctic Grayling (*Thymallus arcticus*).

Supplementary Note 2: Morphological changes during early stages of freshwater adaptation

Some phenotypic divergence between newly founded freshwater Threespine Stickleback populations and their oceanic ancestors are conspicuous[1,6]. One generation after freshwater colonization (i.e., SC2013), the female standard lengths (SL) in Scout Lake declined significantly[7], although SL is phenotypically plastic[8]. Other traits such as lateral plate morph, dorsal fin ray number, and gill raker number are highly heritable[1,9,10].

Specimens for morphological analysis were fixed in 10% buffered formalin, transferred to 50% isopropyl alcohol, and stained in Alizarin Red S, as described in Bell et al. (2004) or Aguirre et al. (2008)[1,10]. We scored three traits, lateral plate morphs and numbers of gill rakers and dorsal fin rays, which differ consistently between anadromous and freshwater stickleback[9] and have diverged rapidly from the ancestral anadromous condition in other lake populations that were found recently by anadromous stickleback[1,4,11].

Standard length is a measure of size often used by fish biologists. It is the distance from the anterior tip of the upper jaw (premaxilla) to the posterior end of the last vertebra (hypural plate). The hypural plate can be located by bending the caudal fin at a 90 degree angle and measuring to the external crease. SL was measured with digital calipers to the nearest 0.1 mm.

Lateral plates are enlarged scales that form a single row along each body side and can be scored with the naked eye. There are three major lateral plate morphs (e.g.,[12,13]) that are strongly influenced by the *Eda* gene[12,14,15]. The complete morph is ancestral to the other two morphs [14]. Anadromous Threespine Stickleback is usually complete morphs[16,17], and the Rabbit Slough population is monomorphic complete[10]. Complete morphs have a plate on each body segment; specimens typically have about 33 plates per side. Complete morphs may be homozygous for the ancestral anadromous allele or heterozygous for it and the derived freshwater allele[14,15,18]. Low morphs usually have four to seven plates per side but no more than 10, which are restricted to the anterior third of the body. Lows are homozygotes for the freshwater allele of *Eda*. Partial morphs have more than 10 anterior plates, an unplated area, and a separate row on the caudal peduncle[12]. They are heterozygous for *Eda*.

Fin rays are jointed bones that support the fins of bony fishes. All fin rays in the dorsal fin were counted under a dissecting microscope. Gill rakers were counted by slitting the membrane at the ventral end of the operculum, and the operculum was lifted to count the number of gill rakers on the anterior edge of the first right gill arch whether they were ossified or not.

We compared the standard length, lateral plate number and dorsal fin ray number between jackpot and non-jackpot individuals in the SC2014 sample and found no significant phenotypic differences between them (Fig. S20). There were 16 out of 21 jackpot carriers that possessed a freshwater allele at the locus that contains the *EDA* gene, which strongly influences the lateral plate morph in Threespine Stickleback[14,15,18] compared to one out of 26 non-jackpot individuals (X^2 (1, N=47) =26.85, *p-value* = 2.197e-07). However, the freshwater alleles in these 16 jackpot carriers were all in a heterozygous state. Thus, they would not be expressed if the allele is

recessive for all phenotypes that it influences[19], and all but one fish (partially plated) had a high plate morph phenotype.

Morphological phenotypes divergent between freshwater and oceanic ecotypes may respond more slowly to directional selection on morphological traits or exert a more significant fitness effect at later stages of adaptation. This situation may explain why traits like loss of armor plating are recessive and likely become expressed after the alleles increase in frequency and are then expressed when individuals inherit two of these recessive alleles in the homozygous state, while freshwater alleles at other loci underlying physiological traits could potentially have higher dominance coefficients such that they can be expressed in the heterozygous state and homozygous state. Indeed, the lack of phenotypic diversity during the early generations may even provide a basis to allow jackpot carriers and non-jackpot individuals to mate freely, with plasticity in traits like body size reducing the response to selection based on size-assortative mating[20,21].

Supplementary Note 3: Origin of jackpot carriers in SC2014

We sought to rule out the unlikely possibility that some resident Threespine Stickleback in Scout Lake survived the rotenone treatment in 2009 and mated with the introduced anadromous stickleback in 2011 to produce the jackpot carriers captured in 2014. Since the previous Threespine Stickleback that inhabited the Scout Lake likely had genotypes similar to nearby established freshwater populations, we retrieved all 51 genomes that were marked freshwater from the Pacific Northeast from ref [24] as a proxy for the previous residents of Scout Lake (see supplementary Data 6 for complete list of genome names). We also used 20 genomes of individuals collected from Rabbit Slough in 2009 to represent the anadromous population that was used to found the lake (Fig. S21A). We then applied our approximate likelihood-based approach (described in the main text) to call genotypes of freshwater adaptive loci for all the 51 freshwater-marked genomes (Fig. S21B). Out of the 51 genomes, we found two with predominantly marine genotypes at these loci despite being marked freshwater. Therefore, we applied a threshold and filtered out all individuals with less than 30% freshwater content at these loci (the minimum proportion of freshwater content we observed in individuals sampled in SC2020). After this filtering, 46 genomes remained. We then created hybrid genotypes from the remaining individuals from the Pacific freshwater populations and the Rabbit Slough individuals and estimated the genotype of the loci (Fig. S22). We found that the individuals had 50 to 63% freshwater content at loci (Fig. S23) compared to the 12.5% to 49.5% content in the individuals we sampled in SC2014. This result suggests that the jackpot carriers sampled in SC2014 were not hybrids from remnant resident freshwater stickleback that might have survived the rotenone treatment. Instead, given that previous studies have identified jackpot carriers in anadromous populations [24,28], jackpot carriers represented in the Scout Lake were descendants of anadromous jackpot carriers from Rabbit Slough.

Supplementary Note 4: Forward-in-time simulations testing jackpot-mediated rapid adaptation

In order to determine if the observed rapid adaptation in Scout Lake is more likely to happen in the presence of jackpot carriers, we performed forward-in-time Wright Fisher simulations to model the process of adaptation with and without jackpot individuals. All simulations were performed using SLiM v5[29,30]. In our simulations, we assume non-overlapping generations, with a generation time of one year. Our simulation framework closely follows that of [24] and [31]. In particular, Kingman et al. were able to use a machine learning inference framework to accurately fit observed allele frequency trajectories at adaptive loci across multiple rapidly adapting freshwater populations, including in Scout Lake. Thus, we base our simulations on the demographic and selective parameters inferred previously in that paper. The beginning of the simulations from [24] capture the early phases of the transporter hypothesis, where freshwater-adaptive alleles circulate between multiple freshwater habitats and the ocean. Accordingly, we simulate a large oceanic (anadromous) population connected to 10 distinct freshwater populations (Fig. S16). We note that we do not simulate linkage amongst loci and thus haploblock structure, which involves a highly complex recombination landscape, but rather we simulated 341 independent loci with freshwater-adaptive alleles. As can be seen in the results below, this approach appears to effectively capture the salient features of rapid adaptation due to jackpot carriers while making the simulations computationally tractable.

We allow migration to occur between freshwater populations and the marine population, but not among freshwater populations. As in Kingman et al. we set the migration rate from marine to freshwater populations (M_AN_TO_FW) to be 0.001, and migration rate from freshwater populations to marine population (M_FW_TO_AN) to be 0.01. These migration rates scale the number of migrants between ocean and freshwater environments such that there are equal migrants to and from each environment.

At the start of the simulation, we introduce freshwater-adaptive mutations at all 341 loci in one of the freshwater populations to mimic an established population that is fully adapted to a freshwater environment. We do not allow de novo mutations during the simulation and each mutation has a selection coefficient of 0.01 in the freshwater environment. The value of 0.01 is of similar magnitude to the mean selection coefficient inferred across loci in Kingman et al. We modeled the fitness of individuals as a quantitative trait under polygenic selection, with an individual's fitness dependent on the proportion of freshwater adaptive alleles they possessed in the freshwater environments. When a mutation enters the oceanic population, its fitness is set as the reciprocal of the corresponding fitness in the freshwater environment. We let the simulation run for 1000 generations, which was enough time for the system to reach equilibrium based on average freshwater content and number of jackpot carriers in each population.

At generation 1000, we established a new freshwater population representing Scout Lake by randomly sampling individuals from the anadromous population. Following its founding, no further migration was permitted between this simulated Scout Lake and any other populations in the system. Individuals with ≥10% of adaptive alleles were designated as jackpot individuals. We then simulated two scenarios: A) Both jackpot carriers and non-jackpot carriers are present in the simulated Scout Lake B) Jackpot carriers are culled from the simulation (by setting their fitness to 0).

We tracked the number of jackpot individuals in the marine population for each of the 1000 generations before the founding of the new lake(Fig. S24). We observed that the proportion of jackpot individuals by 1000 generations was around 1.1%, suggesting that if we found the new lake with 1000 individuals from the marine population, there will be on average 10 jackpot individuals. We varied the founding population sizes for founding the new lake, thereby accounting for the number of jackpot individuals included in the founding population. We used the following as the founding population sizes as: 250, 500, 750, 1000, 2000 and 3000, with the expectation that increasing the founding sizes led to an increased number of jackpot carriers in the newly founded freshwater population. We observed that increasing the founding size indeed increased the number of jackpot carriers (Fig. S17).

We estimated the total freshwater adaptive alleles in the founding population in both scenarios. The results showed that the freshwater content in the two scenarios was not significantly different (KS test: 0.08, p-value:0.908, Fig. 6A), suggesting that the small numbers of jackpot individuals in the founding population did not skew the overall content of freshwater alleles available for adaptation. We then calculated the proportion of freshwater adaptive alleles per individual in the generations corresponding to the sampled timepoints from Scout Lake. We compared these proportions to what we observed from the empirical observation in Scout Lake. For each founding population size (250, 500, 750, 1000, 2000 and 3000), we repeated the simulation 100 times for both scenario A and B.

Supplementary figures

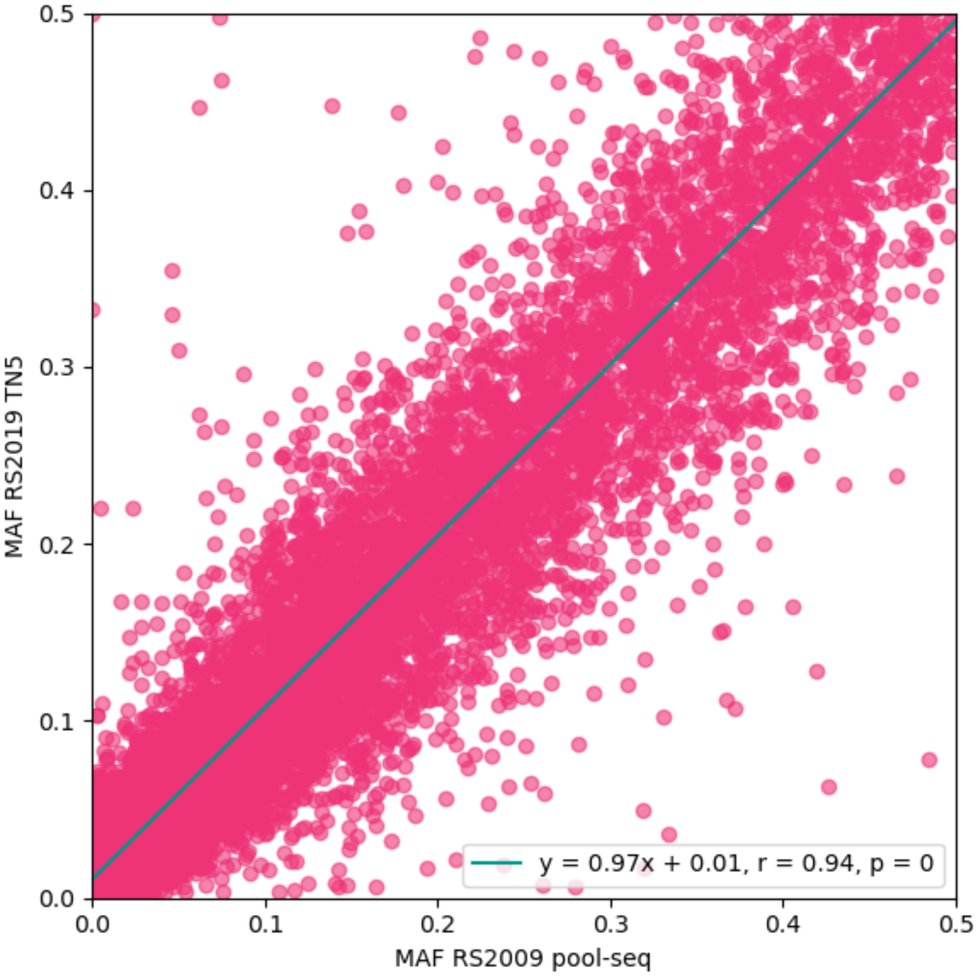

Fig. S1: **Comparing allele frequency estimates from pool-seq and low-coverage.** Correlation between minor allele frequencies (MAF) estimated from a pool-seq of 100 individuals sampled in Rabbit Slough in 2009 and 96 individuals sampled from Rabbit Slough in 2019 used in this study using SNPs from the whole genome.

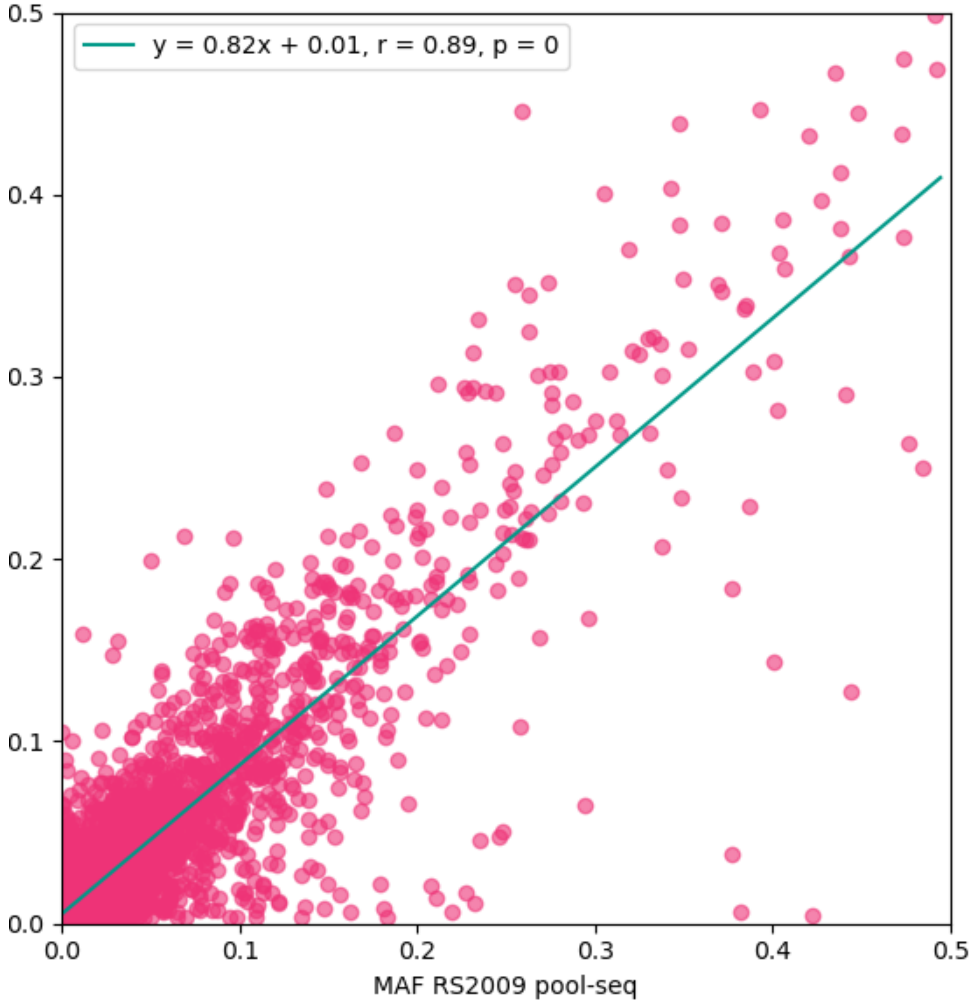

Fig. S2: Comparing allele frequency estimates from pool-seq and low-coverage. Correlation between minor allele frequencies (MAF) estimated from a pool-seq of 100 individuals sampled in Rabbit Slough in 2009 and 96 individuals sampled from Rabbit Slough in 2019 used in this study using SNPs within freshwater adaptive regions.

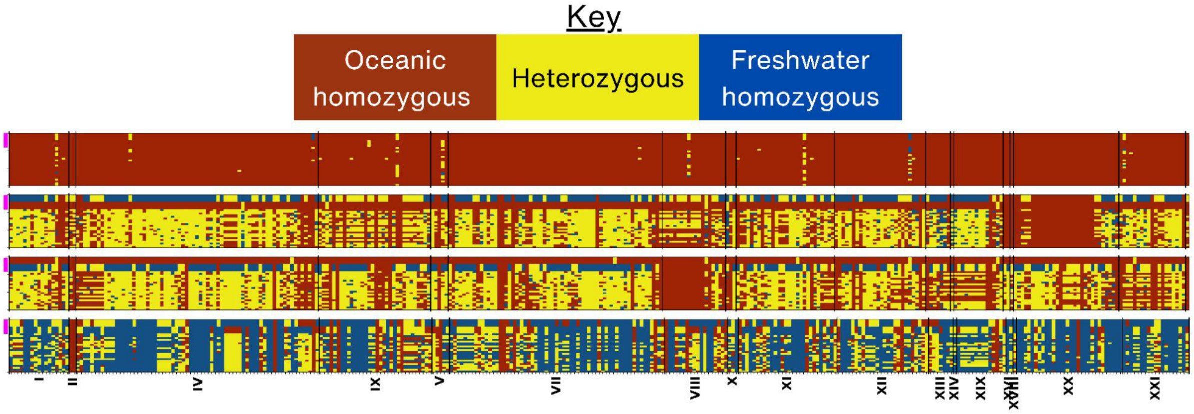

Fig. S3. Haplotypes of crosses used for validating our genotype calling. The crosses were made using stickleback collected from a lake at mile 87 of Seward Highway a few kilometers east of Girdwood, Alaska. The pink rectangles indicate the genomes from parents used for the crosses. The topmost parents are two marine parents followed by their 22 offspring. The next two parents include one marine and one freshwater (one with the male being freshwater and female being marine and vice versa) and their 22 offspring. The bottommost parent are two freshwater individuals, followed by their 22 offspring.

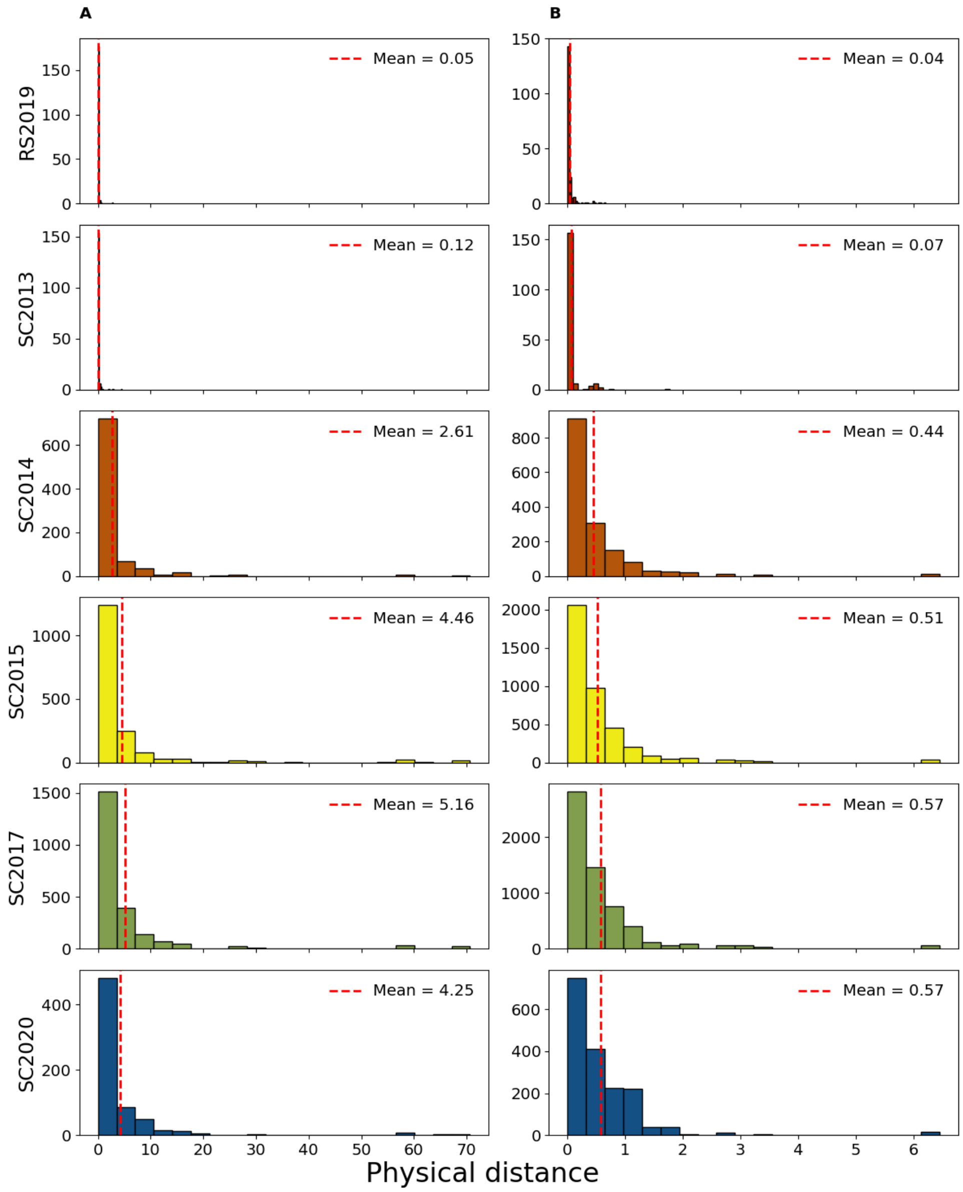

Fig. S4: Comparison of physical distance for each timepoint for different cut off scenarios. A) When there is no threshold (which is reported in the main manuscript) **B)** When the threshold is set to 0.8Mb

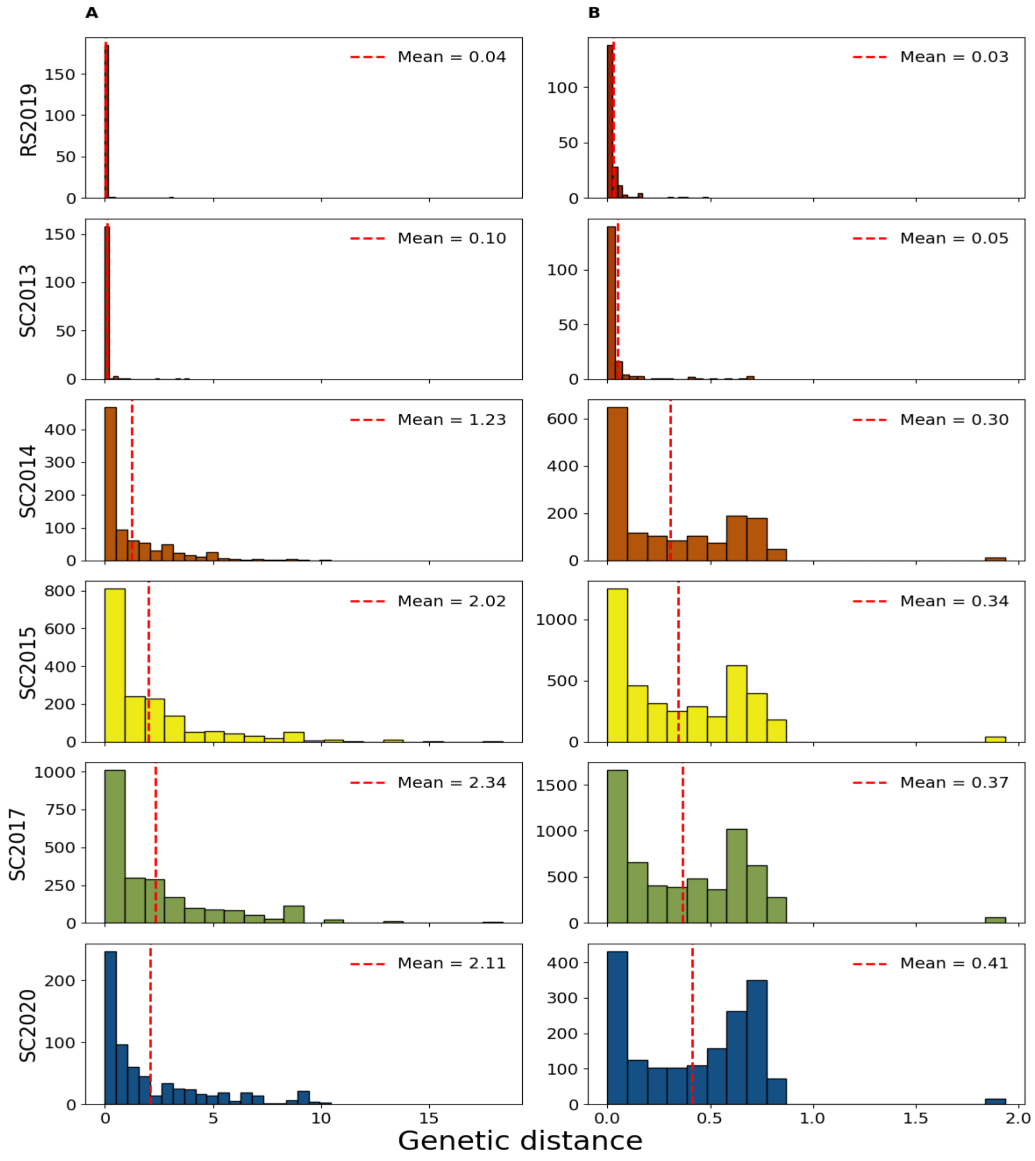

Fig. S5: Comparison of genetic distance for each timepoint for different cut off scenarios. A) When there is no threshold (which is reported in the main manuscript) **B)** When the threshold is set to 0.8Mb

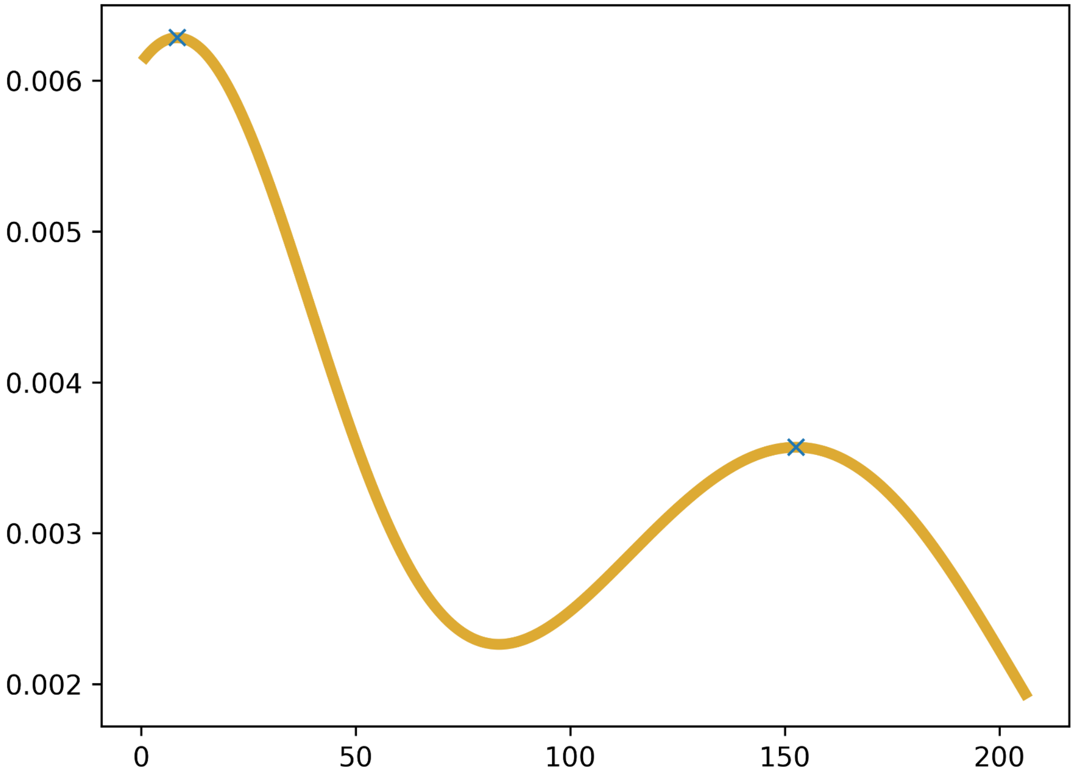

Fig. S6: **Testing for modality of the distribution of freshwater-adaptive alleles**. Dip test showing the two modes of the freshwater adaptive alleles in the SC2014 sample.

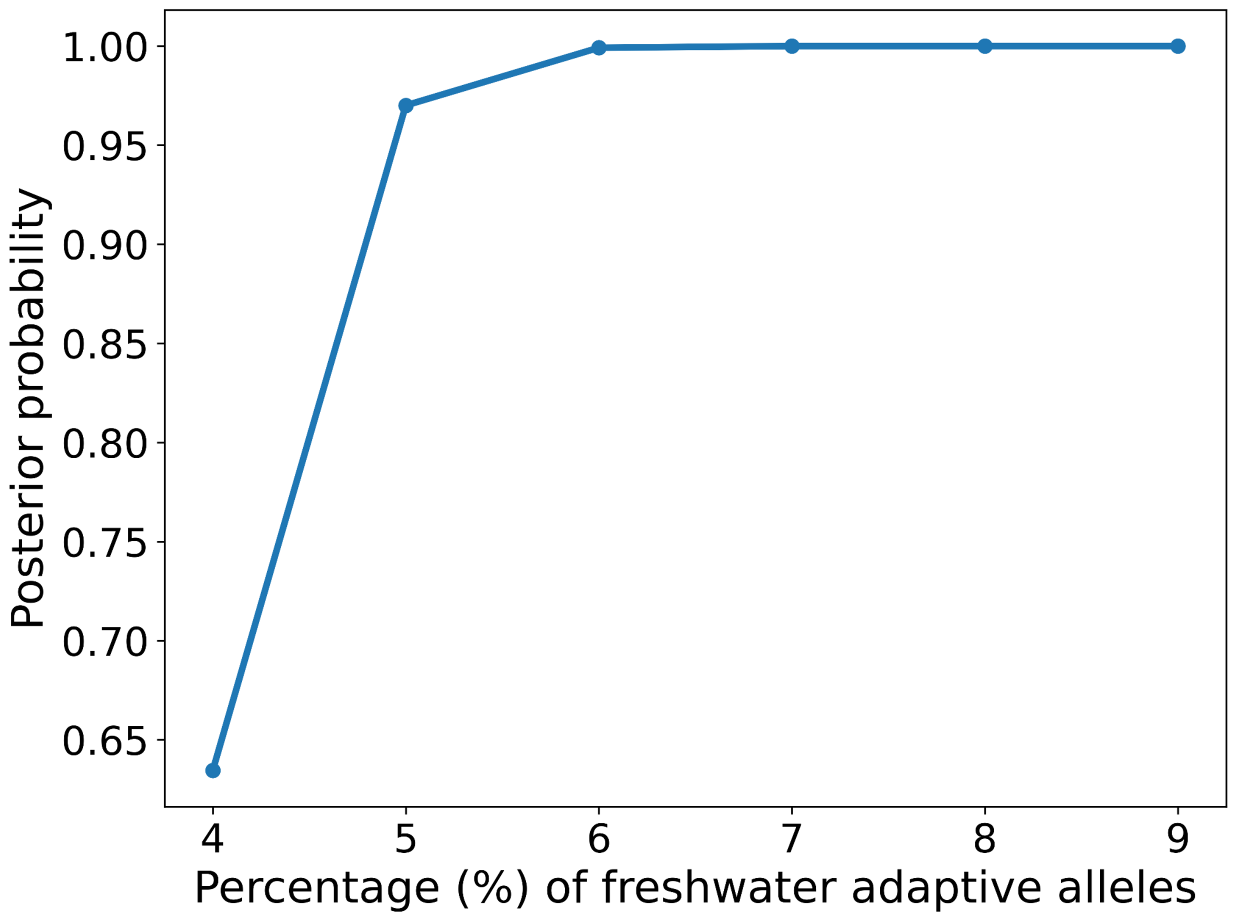

Fig. S7: **Percentage threshold for classifying individuals as jackpot carriers.** Posterior probability of predicting an individual as jackpot or non-jackpot based on the percentage of freshwater adaptive alleles it possesses.

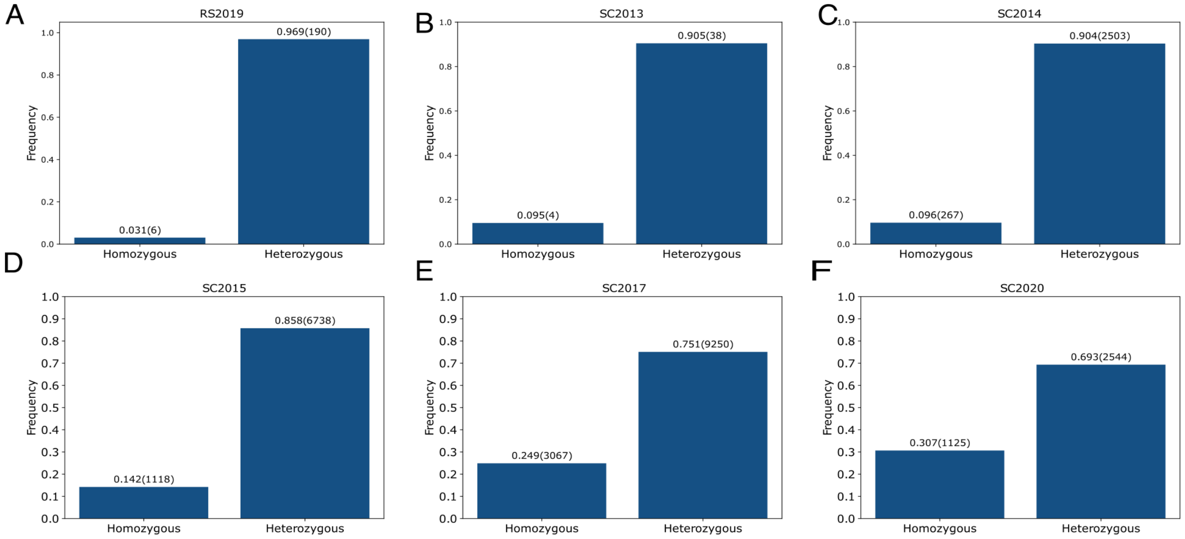

Fig. S8: Heterozygosity over time. Heterozygosity at contiguous blocks of loci involved in freshwater adaptation across the various timepoints from **(A)** RS2009 , **(B)** SC2013, **(C)** SC2014 , **(D)** SC2015 , **(E)** SC2017 and **(F)** SC2020

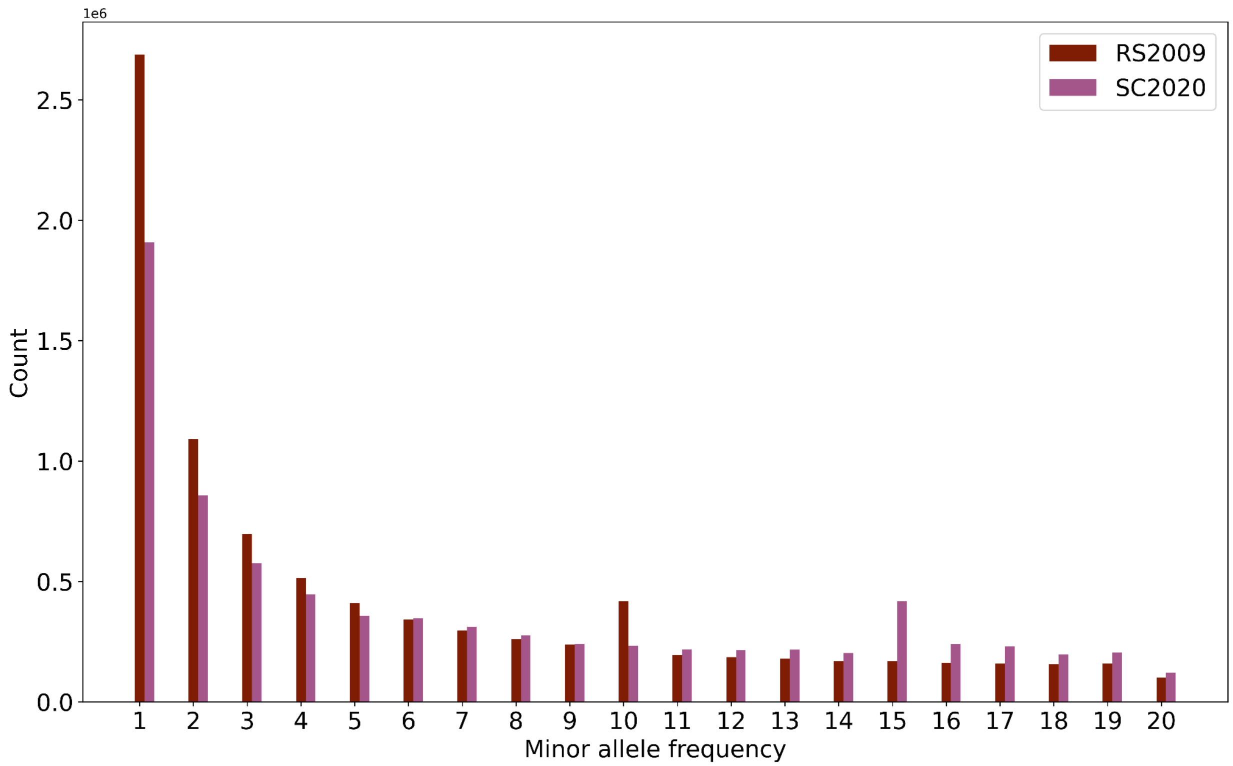

Fig. S9: Site frequency spectrum (SFS) for high coverage genomes. SFS estimated from the whole genome for RS2009 and SC2020. The SFS was folded in dadi and projected down to 40. For clarity, we only show the first 20 polymorphic sites in the SFS.

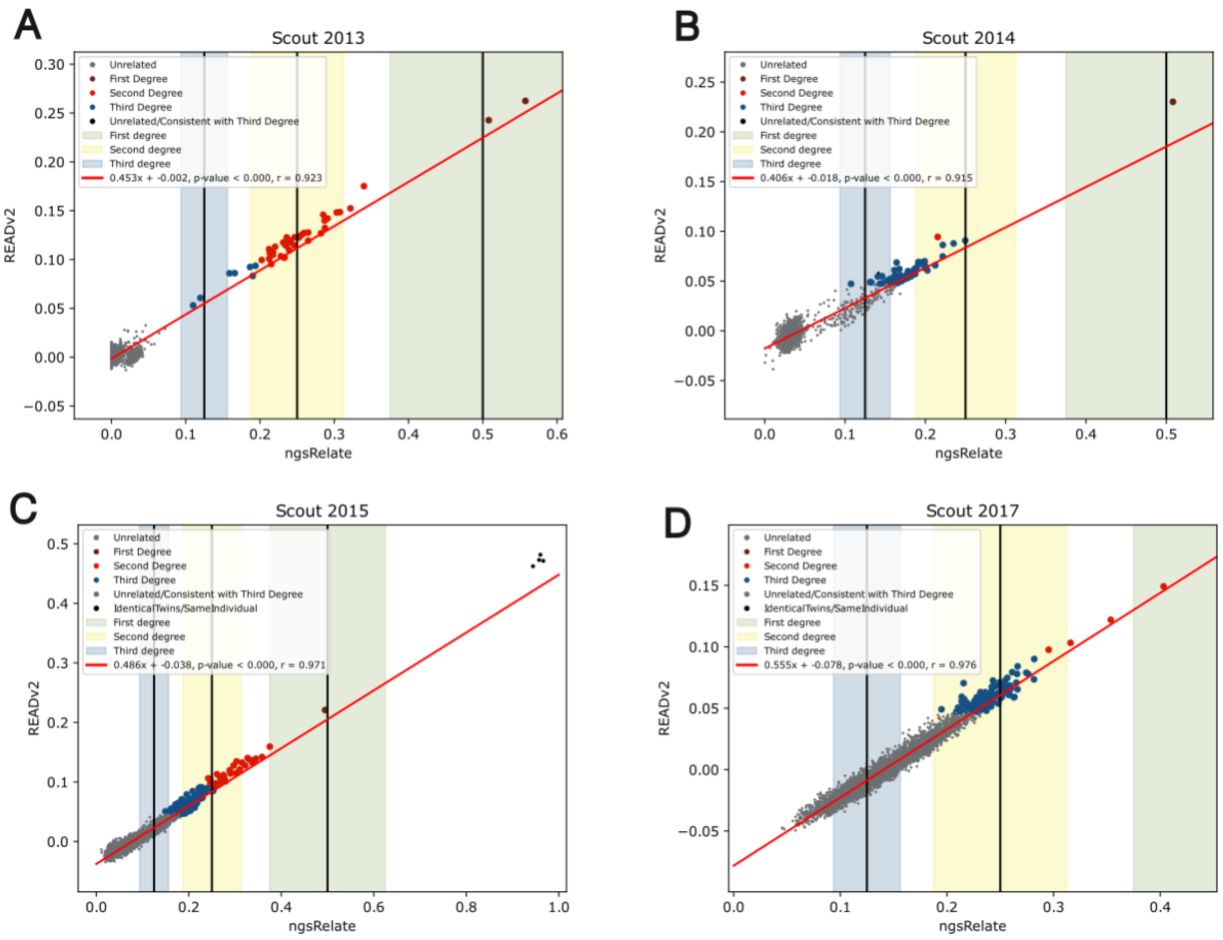

Fig. S10: Relatedness estimates between READv2 and ngRrelate. Correlation of relatedness estimated from READv2[32] and ngsRelate[33] for: A) SC2013. B) SC2014. C) SC2015 D) 2017.

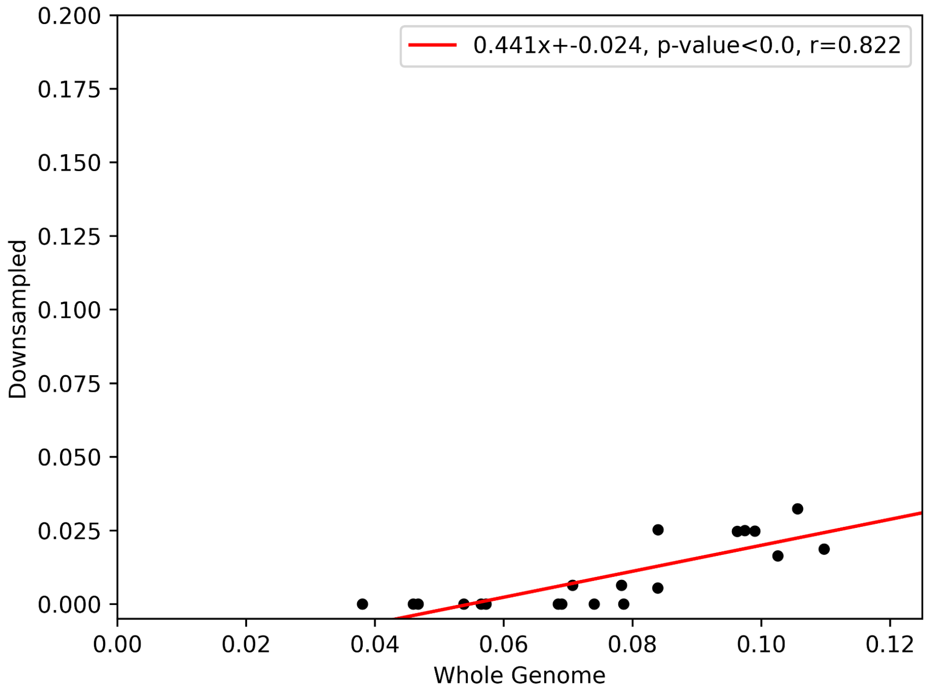

Fig. S11: Comparison of inbreeding coefficients estimated from low- and high-coverage genomes. Inbreeding coefficients estimated from high coverage whole genomes and their downsampled low coverage. We used ngsRelate to estimate the inbreeding coefficient. There is a high correlation (r=0.822) between estimates from downsampled and high coverage specimen

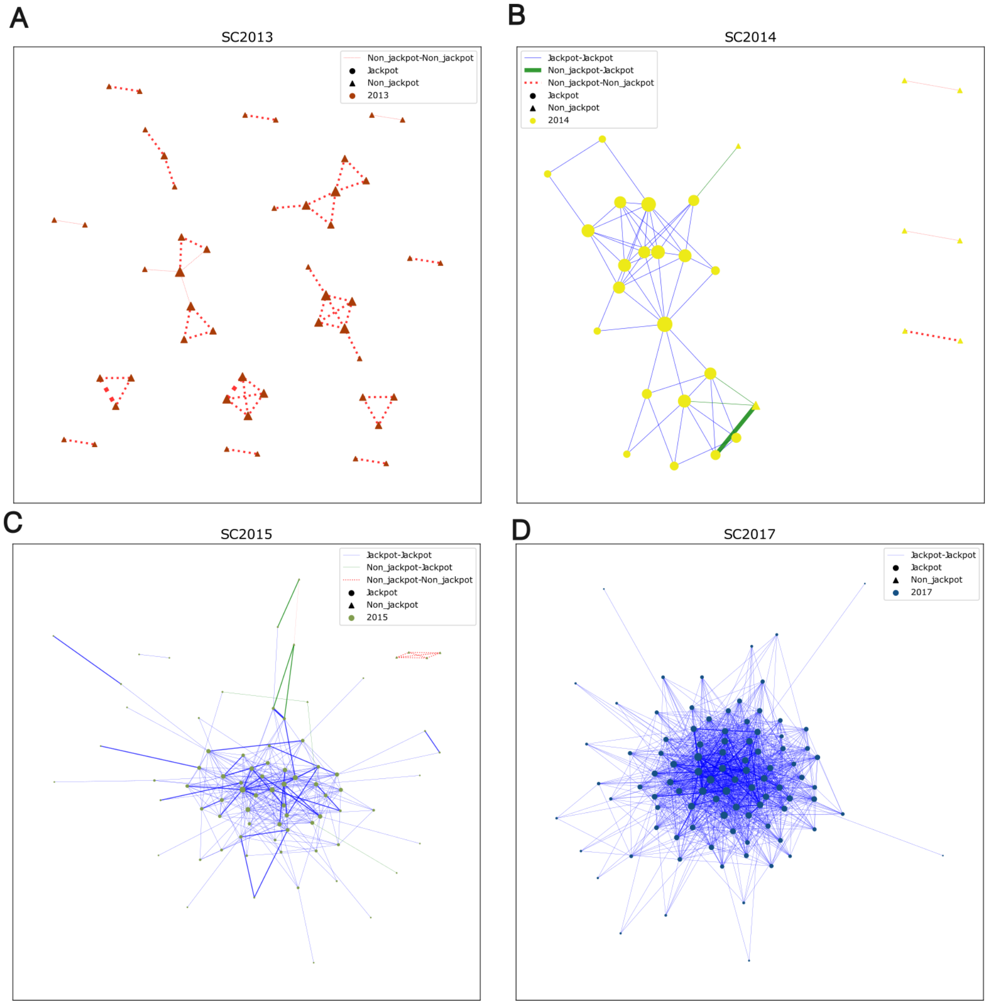

Fig. S12: Intra-year biological relatedness: (A) Relatedness between specimens sampled in SC2013. **(B)** Relatedness between specimens sampled in SC2014. **(C)** Relatedness between specimens sampled in SC2015. **(D)** Relatedness between specimens sampled in SC2017. The thickness of edge reflects the degree of relatedness between the: the thickest edge first-degree, intermediate second-degree and the thinnest edge third-degree relatives. The size of node increases with increasing number of relatives.

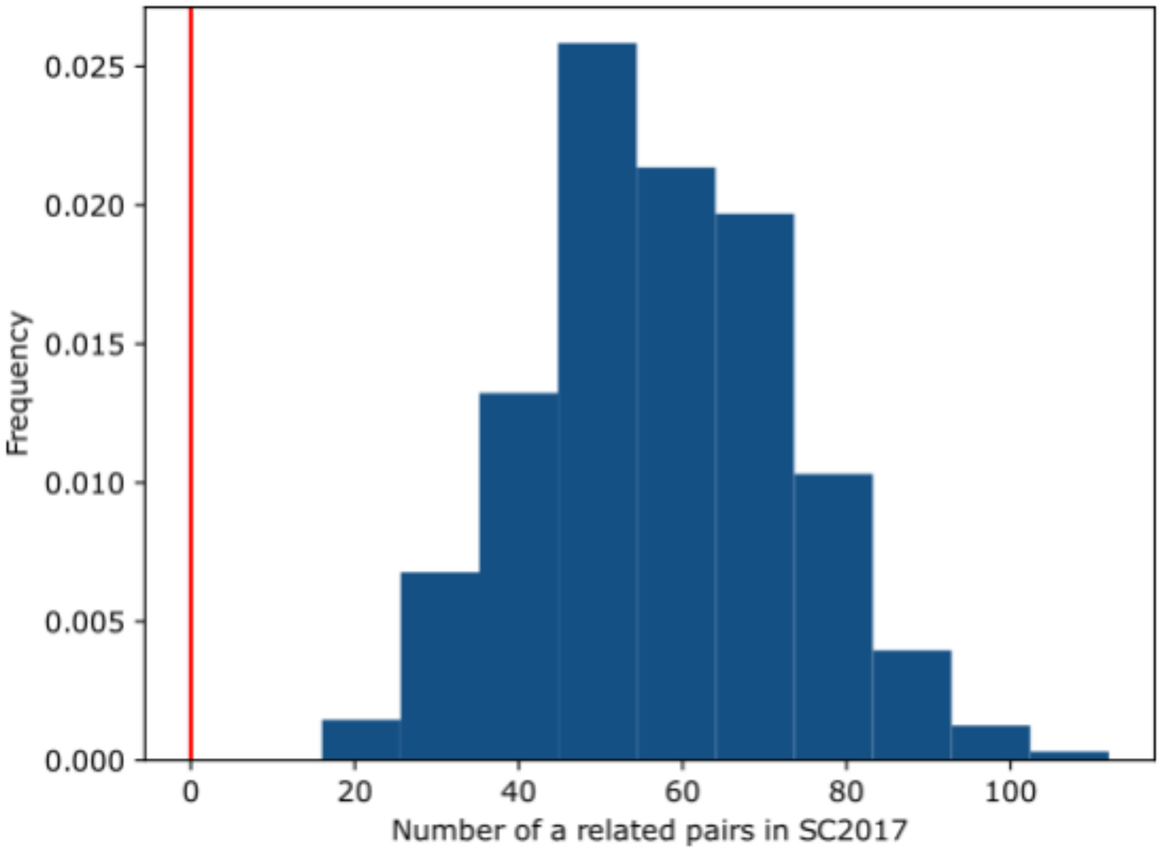

Fig. S13: Permutation of relatedness among SC2017. We randomly sampled 20 individuals from the 96 SC2017 sample 1000 times and observed the number of pairwise relatives of at least third degree. The red vertical line indicates the observed number of relatedness in the SC2020 sample.

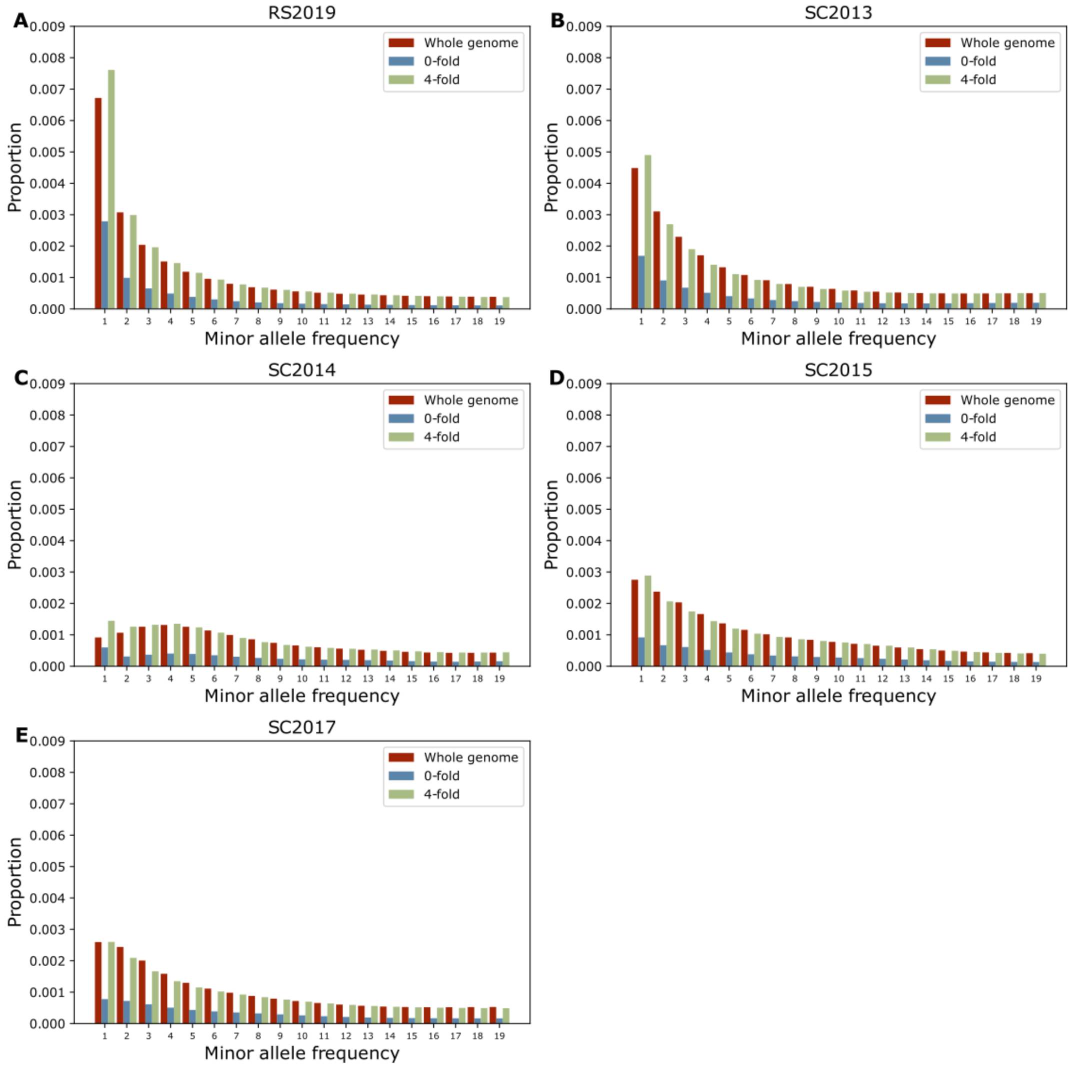

Fig. S14: Site Frequency spectrum at the whole genome, 0- and 4- fold sites for all sampled timepoints except SC2020. (A) RS2019 **(B)** SC2013 **C)** SC2014 **D)** SC2015
E) SC2017.

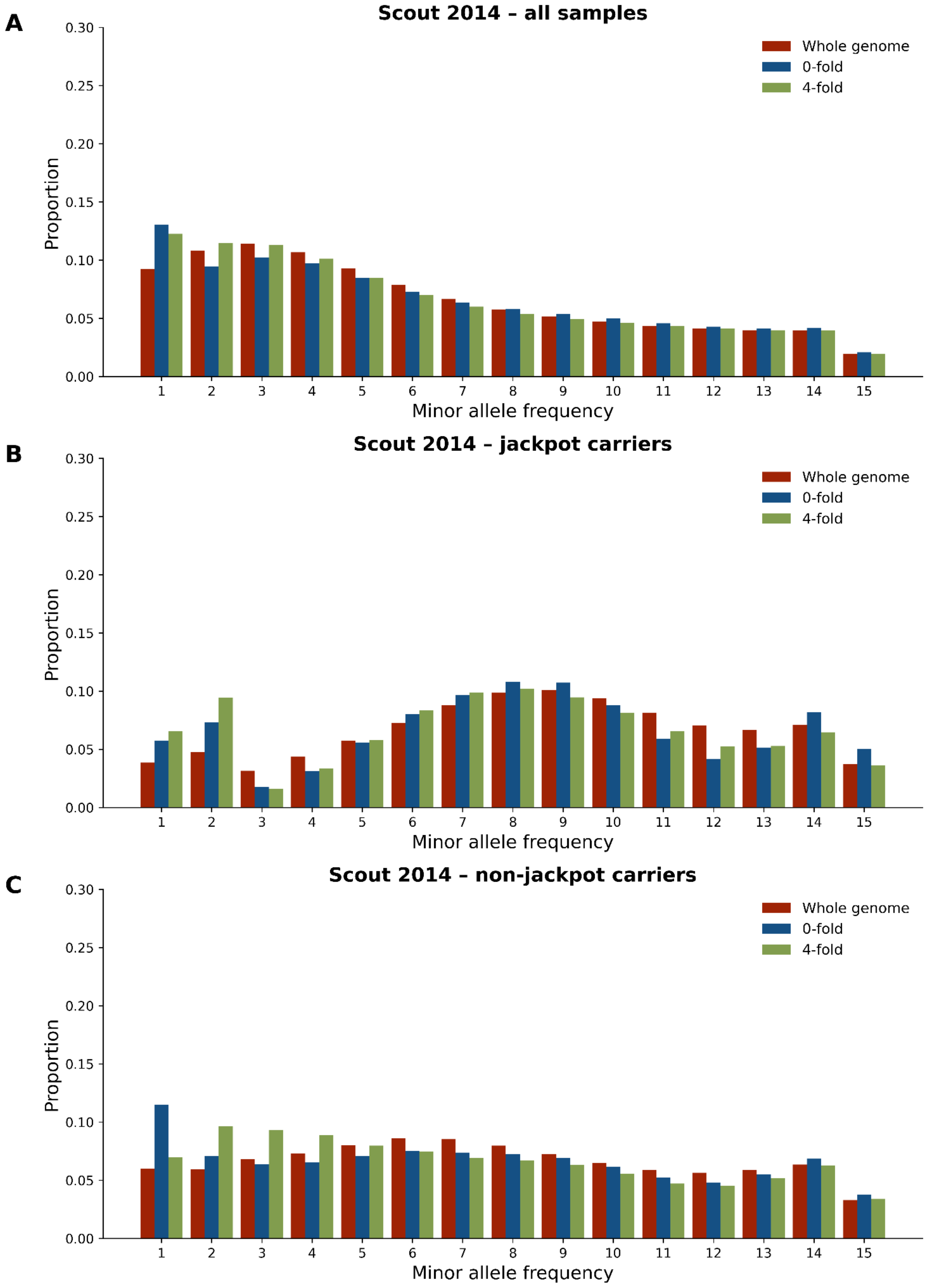

A
Scout 2014 – all samples
B
Scout 2014 – jackpot carriers
C
Scout 2014 – non-jackpot carriers
Whole genome
0-fold
4-fold
Proportion
Minor allele frequency
0.00
0.05
0.10
0.15
0.20
0.25
0.30
1
2
3
4
5
6
7
8
9
10
11
12
13
14
15

Fig. S15: Site frequency spectrum (SFS) of jackpot and non jackpot carriers found in SC2014 at the whole genome, 0-fold and 4-fold sites. (A) SFS for all SC2014 samples combined **(B)** SFS for only jackpot carriers found in SC2014 **(C)** SFS for only non-jackpot individuals found in SC2014.

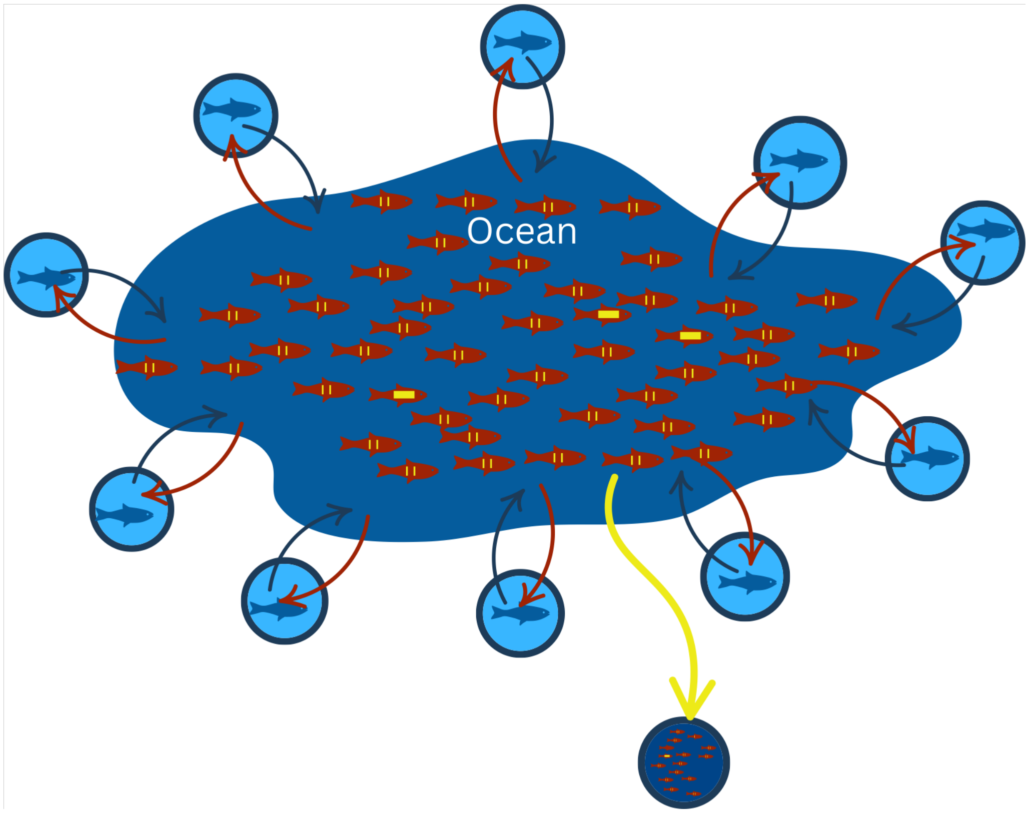

Fig. S16: Set up for SLiM simulations. We simulated a large oceanic (anadromous) population connected to 10 distinct freshwater populations. Migration occurs between freshwater populations and the ocean, but not among freshwater populations. At generation 1000, we founded a new lake with varying population size (see Fig. S17). Figure created by authors using Canva.

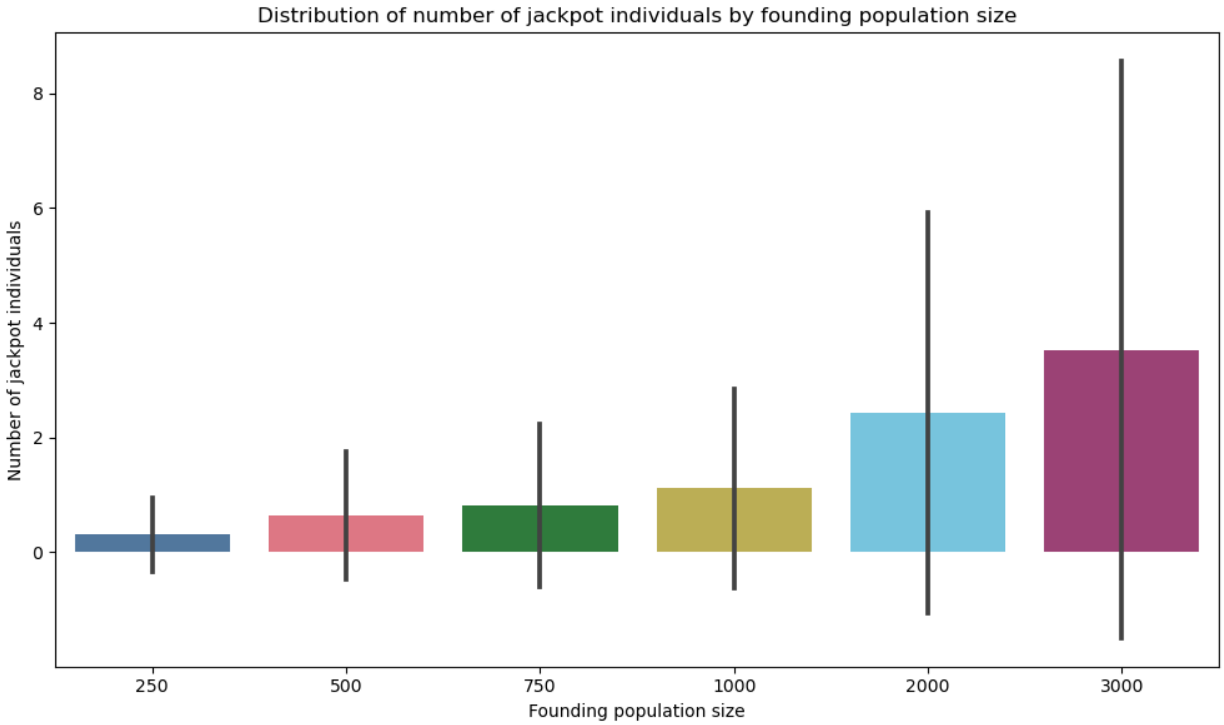

Fig. S17: Distribution of the number of jackpot individuals by founding population size

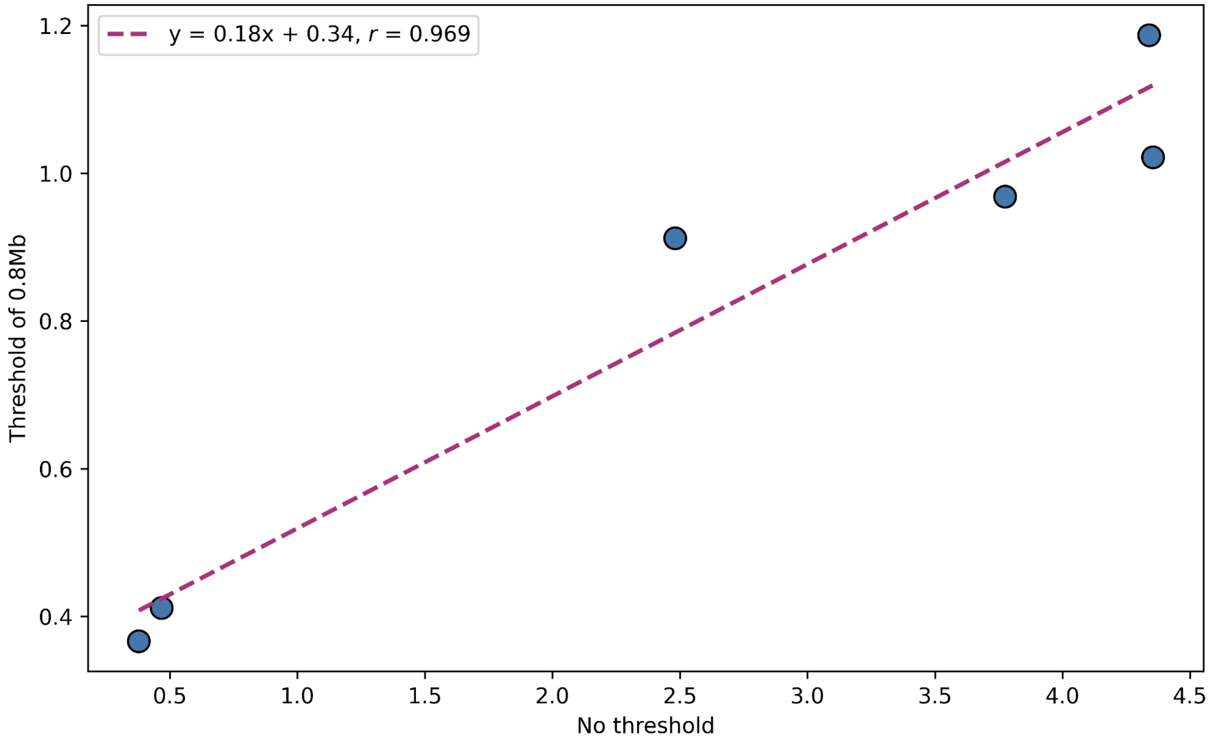

Fig. S18: Plot of average genetic distance when setting a threshold of 0.80Mb (y-axis) and when no threshold is set (x-axis).

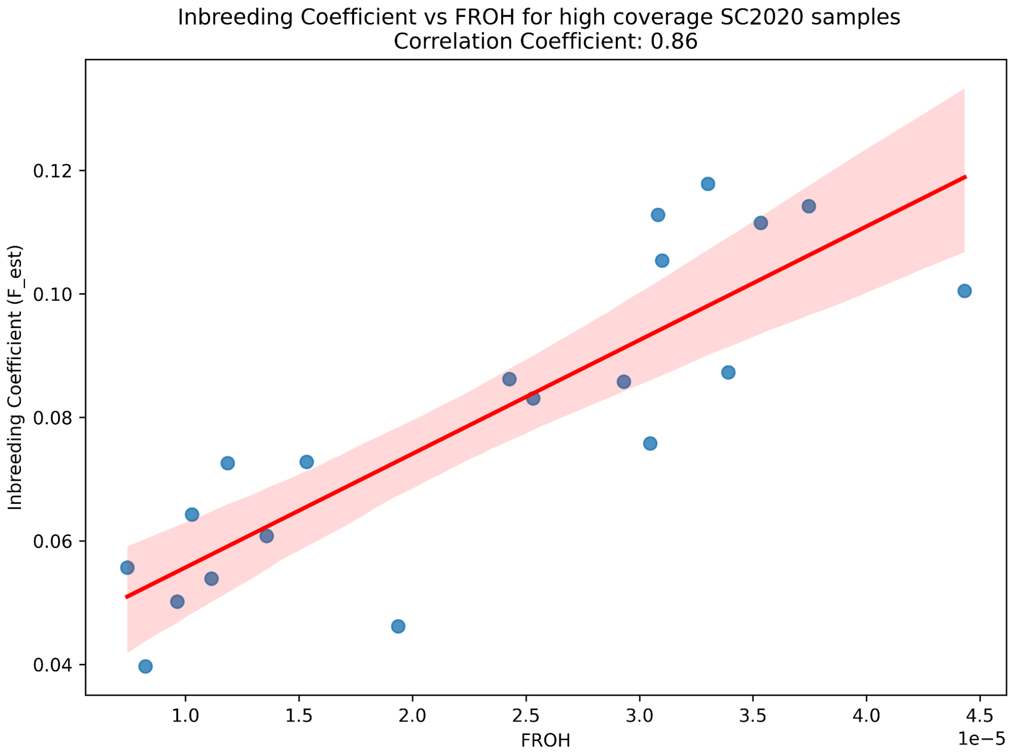

Fig. S19: Comparing runs of homozygosity with inbreeding coefficients. Correlation between estimated Fraction of runs of homozygosity (FROH) and inbreeding coefficients of SC2020 sample.

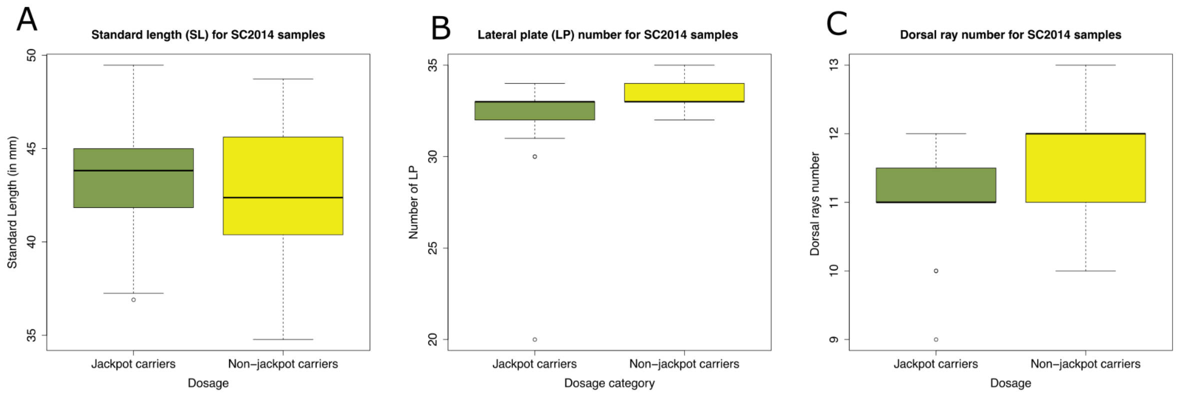

Fig. S20: Morphological phenotypes measured for jackpot carriers and non-jackpot carriers in SC2014. (A) Standard length **(B)** Lateral plate **and (C)** Dorsal ray number

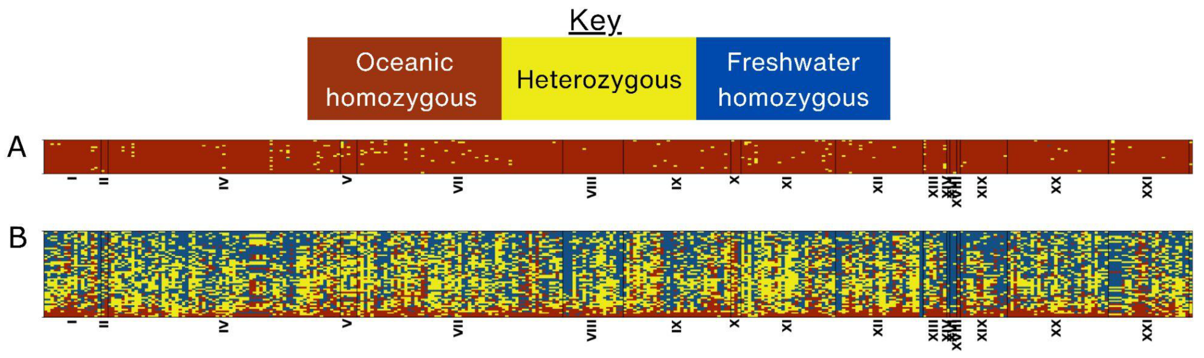

Fig. S21: Genotypes of at freshwater-adaptive loci (A) Rabbit Slough genomes collected in 2009 **(B)** Genomes from freshwater populations in the Pacific from Roberts Kingman et al. Each row is an individual hybrid genome, and each column is one freshwater adaptive loci. A genotype colored red is homozygous for the marine allele, yellow is heterozygous and blue is homozygous for the freshwater allele.

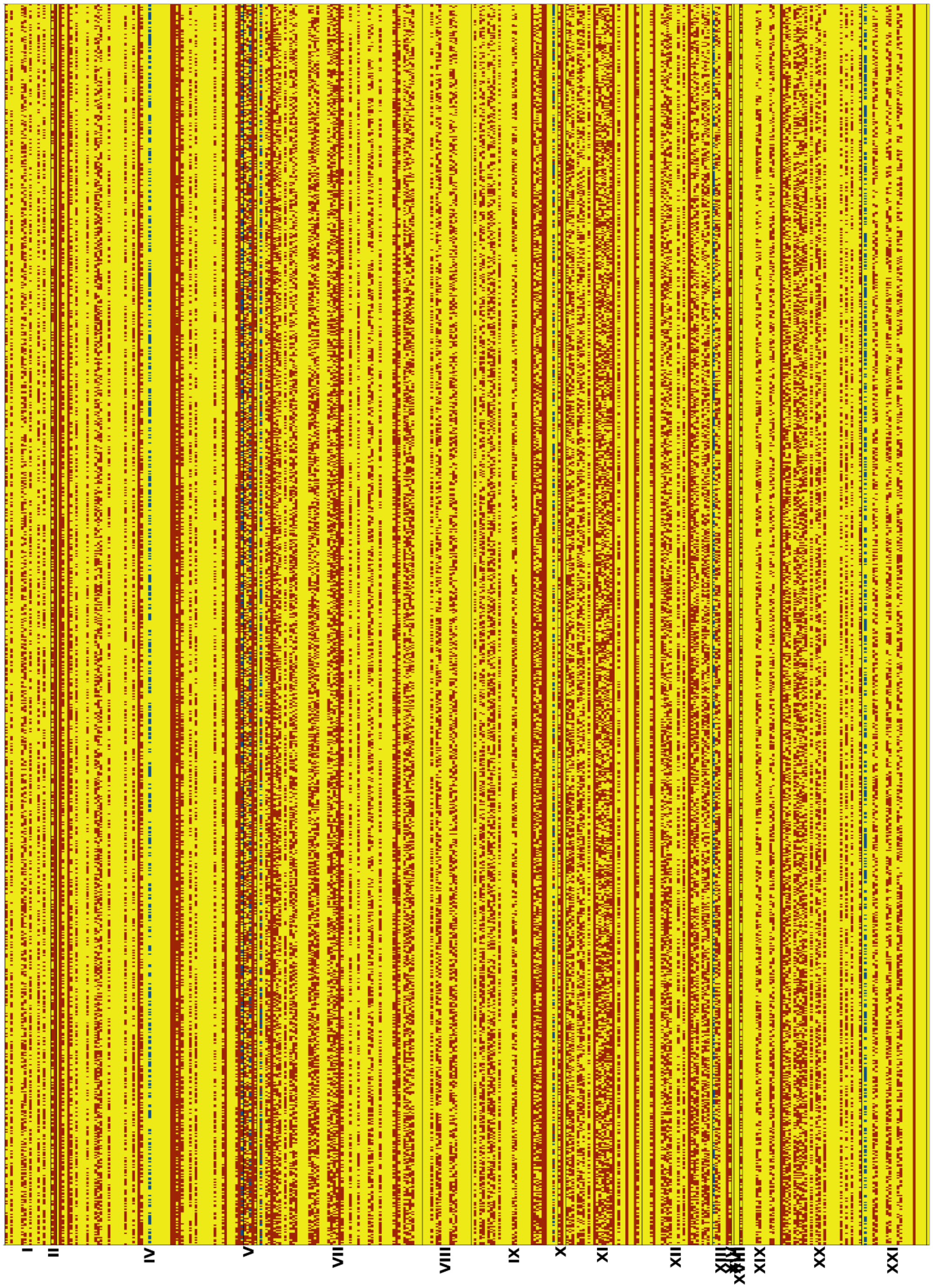

Fig S22: Genotypes of hybrids generated by oceanic individuals and individuals from freshwater populations, as described in SupplementaryNote 3. Each row is an individual hybrid genome and each column is one freshwater adaptive loci. A genotype colored red is homozygous for the marine allele, yellow is heterozygous and blue is homozygous for the freshwater allele.

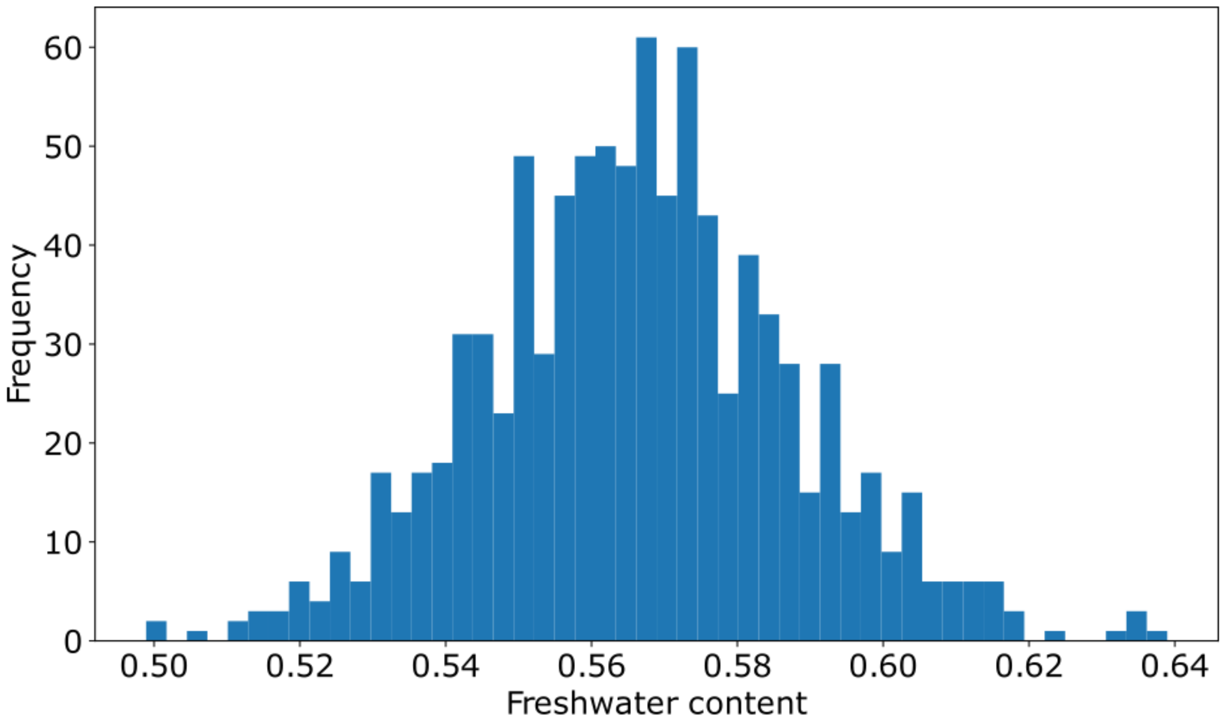

Fig. S23: Distribution of freshwater content of hybrids of oceanic individuals and freshwater individuals

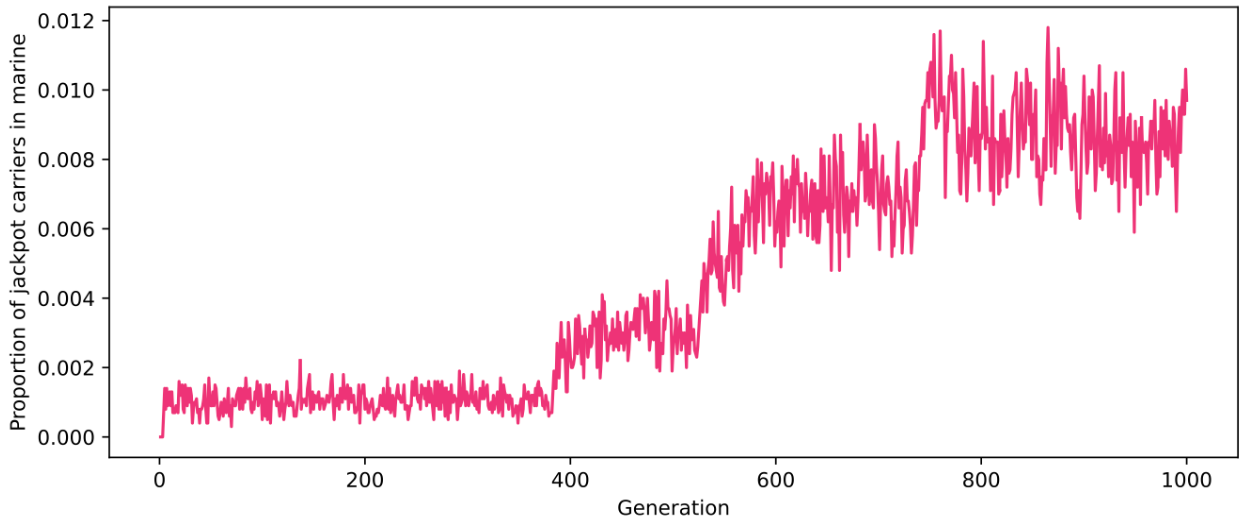

Fig. S24: Number of jackpot carriers in marine population for each generation before founding a new freshwater environment at generation 1000 in a forward-in-time simulation implemented in SLiM.

References

1. Bell, M. A., Aguirre, W. E. & Buck, N. J. Twelve years of contemporary armor evolution in a threespine stickleback population. *Evolution* **58**, 814–824 (2004).
2. Aguirre, W. E. & Bell, M. A. Twenty years of body shape evolution in a threespine stickleback population adapting to a lake environment. *Biol. J. Linn. Soc. Lond.* **105**, 817–831 (2012).
3. Bell, M. A. & Aguirre, W. E. Contemporary evolution, allelic recycling, and adaptive radiation of the threespine stickleback. *Evol. Ecol. Res.* (2013).
4. Klepaker, T. Morphological changes in a marine population of threespined stickleback, Gasterosteus aculeatus, recently isolated in fresh water. *Can. J. Zool.* **71**, 1251–1258 (1993).
5. Bell, M. A. *et al.* Reintroduction of threespine stickleback into Cheney and Scout Lakes, Alaska. *Evol. Ecol. Res.* **17**, 157–178 (2016).
6. Bell, M. A. & Foster, S. A. Introduction to the evolutionary biology of the threespine stickleback. *The evolutionary biology of the threespine* (1994).
7. Kurz, M. L., Heins, D. C., Bell, M. A. & von Hippel, F. A. Shifts in life-history traits of two introduced populations of threespine stickleback. *Evol. Ecol. Res.* **17**, 225–242 (2016).
8. Wund, M. A., Singh, O. D., Geiselman, A. & Bell, M. A. Morphological evolution of an anadromous threespine stickleback population within one generation after reintroduction to Cheney Lake, Alaska. *Evolutionary Ecology Research* **17**, 203–224 (2016).
9. McPhail, J. D. Speciation and the evolution of reproductive isolation in the sticklebacks (*Gasterosteus*) of south-western British Columbia. in *The Evolutionary Biology of the Threespine Stickleback* 399–437 (Oxford University PressOxford, 1994).
10. Aguirre, W. E., Ellis, K. E., Kusenda, M. & Bell, M. A. Phenotypic variation and sexual dimorphism in anadromous threespine stickleback: implications for postglacial adaptive radiation. *Biol. J. Linn. Soc. Lond.* **95**, 465–478 (2008).
11. Aguirre, W. E. *et al.* Freshwater Colonization, Adaptation, and Genomic Divergence in Threespine

Stickleback. *Integr. Comp. Biol.* **62**, 388–405 (2022).

12. Bell, M. A. Lateral plate polymorphism and ontogeny of the complete plate morph of threespine sticklebacks (Gasterosteus aculeatus). *Evolution* **35**, 67–74 (1981).
13. Hagen, D. W. & Gilbertson, L. G. Geographic variation and environmental selection in Gasterosteus aculeatus l. In the pacific northwest, America. *Evolution* **26**, 32–51 (1972).
14. Colosimo, P. F. *et al.* Widespread parallel evolution in sticklebacks by repeated fixation of Ectodysplasin alleles. *Science* **307**, 1928–1933 (2005).
15. Cresko, W. A. *et al.* Parallel genetic basis for repeated evolution of armor loss in Alaskan threespine stickleback populations. *Proc. Natl. Acad. Sci. U. S. A.* **101**, 6050–6055 (2004).
16. Hagen, D. W. Isolating mechanisms in threespine sticklebacks (Gasterosteus). *J. Fish. Res. Board Can.* **24**, 1637–1692 (1967).
17. Klepaker, T. Lateral plate polymorphism in marine and estuarine populations of the threespine stickleback (Gasterosteus aculeatus) along the coast of Norway. *Copeia* **1996**, 832 (1996).
18. Colosimo, P. F. *et al.* The genetic architecture of parallel armor plate reduction in threespine sticklebacks. *PLoS Biol.* **2**, E109 (2004).
19. Albert, A. Y. K. *et al.* The genetics of adaptive shape shift in stickleback: pleiotropy and effect size. *Evolution* **62**, 76–85 (2008).
20. Baker, J. A. *et al.* Life-history plasticity in female threespine stickleback. *Heredity* **115**, 322–334 (2015).
21. Rollins, J. L., Chiang, P., Waite, J. N., von Hippel, F. A. & Bell, M. A. Jacks and Jills: alternative life-history phenotypes and skewed sex ratio in anadromous Threespine Stickleback (Gasterosteus aculeatus). *Evol. Ecol. Res.* **18**, 363–382 (2017).
22. Baer, J., Ziegaus, S., Schumann, M., Geist, J. & Brinker, A. Escaping malnutrition by shifting habitats: A driver of three-spined stickleback invasion in Lake Constance. *J. Fish Biol.* **104**, 746–757 (2024).
23. Ishikawa, A. *et al.* A key metabolic gene for recurrent freshwater colonization and radiation in

fishes. *Science* **364**, 886–889 (2019).

24. Roberts Kingman, G. A. *et al.* Predicting future from past: The genomic basis of recurrent and rapid stickleback evolution. *Sci Adv* **7**, (2021).
25. McCairns, R. J. S. & Bernatchez, L. Adaptive divergence between freshwater and marine sticklebacks: Insights into the role of phenotypic plasticity from an integrated analysis of candidate gene expression. *Evolution* **64**, (2009).
26. Kusakabe, M., Mori, S. & Kitano, J. Gill Na+/K+-ATPase in the threespine stickleback (Gasterosteus aculeatus): changes in transcript levels and sites of expression during acclimation to seawater. *Evolutionary Ecology Research* **20**, 349–363 (2019).
27. Divino, J. N. *et al.* Osmoregulatory physiology and rapid evolution of salinity tolerance in threespine stickleback recently introduced to fresh water. *Evol. Ecol. Res.* **17**, 179–201 (2016).
28. Bassham, S., Catchen, J., Lescak, E., von Hippel, F. A. & Cresko, W. A. Repeated Selection of Alternatively Adapted Haplotypes Creates Sweeping Genomic Remodeling in Stickleback. *Genetics* **209**, 921–939 (2018).
29. Haller, B. C. & Messer, P. W. SLiM 4: Multispecies Eco-evolutionary modeling. *Am. Nat.* **201**, E127–E139 (2023).
30. Haller, B. C., Ralph, P. L. & Messer, P. W. SLiM 5: Eco-evolutionary simulations across multiple chromosomes and full genomes. *bioRxiv* (2025) doi:10.1101/2025.08.07.669155.
31. Galloway, J., Cresko, W. A. & Ralph, P. A Few Stickleback Suffice for the Transport of Alleles to New Lakes. *G3* **10**, 505–514 (2020).
32. Alaçamlı, E. *et al.* READv2: Advanced and user-friendly detection of biological relatedness in archaeogenomics. *bioRxiv* 2024.01.23.576660 (2024) doi:10.1101/2024.01.23.576660.
33. Hanghøj, K., Moltke, I., Andersen, P. A., Manica, A. & Korneliussen, T. S. Fast and accurate relatedness estimation from high-throughput sequencing data in the presence of inbreeding. *Gigascience* **8**, (2019).
