# Supplementary material for: Rare jackpot individuals drive rapid adaptation in Threespine Stickleback
Source: Nat Commun. 2026 Mar 30;17:4614. doi: 10.1038/s41467-026-71236-y (PMC13199453; doi:10.1038/s41467-026-71236-y)
Supplement: Supplementary file 2 — Descriptions of Additional Supplementary Files [file 41467_2026_71236_MOESM2_ESM.pdf]

Description of Additional Supplementary Files

Supplementary Data 1: Sample sizes, average sequencing coverage, and count of jackpot individuals across all time points.

Supplementary Data 2: Distribution of defined haploblocks of the freshwater adaptive loci across Scout Lake samples. For each sample, the genotype of each freshwater adaptive locus (FWAL) that make up the contiguous haploblock, the chromosome that the haploblock can be found, the genomic coordinate for each of the haploblocks, and the cumulative size of the entire contiguous haploblock is provided in columns B-E respectively.

Supplementary Data 3: Genomes used for the construction of a reference panel for imputation of the low coverage dataset. The location the sample was collected, the ecotype (we use experimental to indicate samples collected from lakes recently founded by transplanting anadromous fish into the lakes(Aguirre et al. 2022; Bell et al. 2016)), the coverage of the genome and the study that originally published the genomes.

Supplementary Data 4: Pairwise relatedness between all samples collected in Scout Lake used in this study (SC2013, SC2014, SC2015, SC2017 and SC2020) as well as RS2019 samples. We provide relatedness estimates from both READv2(Alaçamlı et al. 2024) and ngsRelate(Hanghøj et al. 2019).

Supplementary Data 5: Loci with adaptive alleles that rapidly increase in new freshwater environments as identified by(Roberts Kingman et al. 2021). For each loci, we provide the number of SNPs within each region, the freshwater and marine alleles at each SNP.

Supplementary Data 6: Details of genomes of putatively freshwater samples from(Roberts Kingman et al. 2021) that were used in supplementary section 14.
