# Supplementary material for: Rare jackpot individuals drive rapid adaptation in Threespine Stickleback
Source: Nat Commun. 2026 Mar 30;17:4614. doi: 10.1038/s41467-026-71236-y (PMC13199453; doi:10.1038/s41467-026-71236-y)
Supplement: Supplementary file 9 — Reporting Summary [file 41467_2026_71236_MOESM9_ESM.pdf]

nature portfolio

Corresponding author(s): Krishna R. Veeramah

Last updated by author(s): Jan 23, 2026

Reporting Summary

Nature Portfolio wishes to improve the reproducibility of the work that we publish. This form provides structure for consistency and transparency in reporting. For further information on Nature Portfolio policies, see our Editorial Policies and the Editorial Policy Checklist.

Statistics

For all statistical analyses, confirm that the following items are present in the figure legend, table legend, main text, or Methods section.

n/a	Confirmed	
☐	☒	The exact sample size (*n*) for each experimental group/condition, given as a discrete number and unit of measurement
☒	☐	A statement on whether measurements were taken from distinct samples or whether the same sample was measured repeatedly
☐	☒	The statistical test(s) used AND whether they are one- or two-sided<br>*Only common tests should be described solely by name; describe more complex techniques in the Methods section.*
☒	☐	A description of all covariates tested
☐	☒	A description of any assumptions or corrections, such as tests of normality and adjustment for multiple comparisons
☐	☒	A full description of the statistical parameters including central tendency (e.g. means) or other basic estimates (e.g. regression coefficient) AND variation (e.g. standard deviation) or associated estimates of uncertainty (e.g. confidence intervals)
☐	☒	For null hypothesis testing, the test statistic (e.g. *F*, *t*, *r*) with confidence intervals, effect sizes, degrees of freedom and *P* value noted<br>*Give P values as exact values whenever suitable.*
☒	☐	For Bayesian analysis, information on the choice of priors and Markov chain Monte Carlo settings
☒	☐	For hierarchical and complex designs, identification of the appropriate level for tests and full reporting of outcomes
☐	☒	Estimates of effect sizes (e.g. Cohen's *d*, Pearson's *r*), indicating how they were calculated

Our web collection on statistics for biologists contains articles on many of the points above.

Software and code

Policy information about availability of computer code

Data collection	Not applicable
Data analysis	All custom scripts have been deposited to GitHub and can be accessed through the following link: https://github.com/a-kwakye/Rare-Jackpot-Individuals-Drive-Rapid-Adaptation-in-Threespine-Stickleback and code ocean at https://codeocean.com/capsule/9257557/tree. We used the following softwares in our analyses and have been appropriately mentioned in the main manuscript<br><br>AdapterRemoval (ver. 2.2.2)<br>GATK version 3.7<br>Beagle 4.0<br>angsd<br>Degenerate v1.2.1<br>dadi<br>READv2<br>NGSrelate<br>PLINK 2.0<br>samtools

For manuscripts utilizing custom algorithms or software that are central to the research but not yet described in published literature, software must be made available to editors and reviewers. We strongly encourage code deposition in a community repository (e.g. GitHub). See the Nature Portfolio guidelines for submitting code & software for further information.

Data

Policy information about availability of data

All manuscripts must include a data availability statement. This statement should provide the following information, where applicable:

- Accession codes, unique identifiers, or web links for publicly available datasets
- A description of any restrictions on data availability
- For clinical datasets or third party data, please ensure that the statement adheres to our policy

The whole genome data generated in this study have been deposited in the Sequence Read Archive (www.ncbi.nlm.nih.gov/sra) under accession code PRJNA1231081[https://www.ncbi.nlm.nih.gov/sra/?term=PRJNA1231081]. Whole genomes from Rabbit Slough previously published in 12 can be found at SRA under accession code PRJNA671690[https://www.ncbi.nlm.nih.gov/sra/?term=PRJNA671690]. All other whole genomes from 12 can be found under accession code PRJNA247503[https://www.ncbi.nlm.nih.gov/sra/?term=PRJNA247503]. Source data are provided as a Source Data file.

Research involving human participants, their data, or biological material

Policy information about studies with human participants or human data. See also policy information about sex, gender (identity/presentation), and sexual orientation and race, ethnicity and racism.

Reporting on sex and gender	Not applicable
Reporting on race, ethnicity, or other socially relevant groupings	Not applicable
Population characteristics	Not applicable
Recruitment	Not applicable
Ethics oversight	Not applicable

Note that full information on the approval of the study protocol must also be provided in the manuscript.

Field-specific reporting

Please select the one below that is the best fit for your research. If you are not sure, read the appropriate sections before making your selection.

☐ Life sciences ☐ Behavioural & social sciences ☒ Ecological, evolutionary & environmental sciences

For a reference copy of the document with all sections, see nature.com/documents/nr-reporting-summary-flat.pdf

Ecological, evolutionary & environmental sciences study design

All studies must disclose on these points even when the disclosure is negative.

Study description	We sequenced 452 whole genomes from samples collected annually, spanning the first few generations of rapid adaptation to conditions in Scout Lake as well as samples from the ancestral population that was used to found the Scout Lake population.
Research sample	This study used samples of Gasteroteus aculeatus (Threespine Stickleback) that were collected annually. The samples include both males and females. Minnow traps, which can only catch adult fish were used in sampling, therefore the individuals collected were likely more than 6 months year old. We can confirm that all samples were collected following ethical approval from Stony Brook University and The College of New Jersey Institutional Care and Use of Animals Committee.
Sampling strategy	We did not perform any statistical methods to predetermine sample size. Our study relies predominantly on previously identified variants, therefore we did not require sample size calculations. However, our sample sizes of 96 (and 48 for SC2014; 20 for SC2020) provide sufficient power to detect even low frequency genetic variants.
Data collection	Minnow traps with a mesh of 6.35 mm or usually 3.175 mm have been set yearly for up to 24 hours at less than 2 m depth and 5 m from shore. After capture, TS are separated from other fish species and transferred to a bucket of lake water to which equal volumes of sodium bicarbonate and MS-222 (tricaine methane-sulfonate) had been added at a high enough concentration to cause the fish to lose equilibrium within 30 seconds and to die within a few minutes. Death was inferred from the failure of the fish to react to tapping the side of the bucket in which they were held and then to pinching the caudal fins of selected fish. We netted the fish out of the bucket, washed them in lake water, and dropped them into 70 % ethanol in deionized water in a 1-liter bottle with up to about half fish and the remainder with ethanol solution. The ethanol was replaced with a fresh 70% ethanol solution within about 24 hours after the lipid from the fish had discolored the ethanol with a yellow hue. The right pectoral fin was usually clipped from the specimen and placed into a small, numbered conical tube of 70% ethanol for DNA extraction, and the remainder of the fish was placed with another fish from the same sample in a numbered, 15-ml tube with 70% ethanol and one fish head up and the other head down.

	These fish are stored either in David M. Kingsley's laboratory in the Department of Developmental Biology, Stanford School of Medicine or in KRV's lab. All procedures to introduce and sample TS from Scout Lake were approved by the Institutional Animal Use and Care Committee at Stony Brook University to MAB or at Stanford University to David M. Kingsley, and stickleback were collected under annual Fish Resource Permits from the Alaska Department of Fish and Game to MAB. Sampling were done by MAB and DCH.
Timing and spatial scale	Samples of Threespine Stickleback have been collected from Scout Lake at least once a year around the start of the breeding season in late May or June since 2012.
Data exclusions	No data generated were excluded from analyses, except four genomes from SC2015 that our kinship analyses predicted to be likely to be duplicates. We removed one individual of the pair in all related analyses.
Reproducibility	To ensure the conclusions from this study are easily reproduced, we have deposited all the genomic datasets we generated in SRA. We have also made all relevant scripts publicly available as well as added pertinent data for generating the main figures as source data.
Randomization	we randomly selected 96 specimens with high concentrations of DNA for each time point except SC2014 sample where we only had 48 available, in which case we utilized all available specimen.
Blinding	Blinding was not necessary for DNA quantification, library preparation. In addition, no blinding was necessary for bioinformatic analyses as they were performed using standardized computational approaches.

Did the study involve field work? ☒ Yes ☐ No

Field work, collection and transport

Field conditions	Samples were taken around May/June.
Location	Scout Lake (60.5353N, 150.8322W) is on the Kenai Peninsula, Alaska, USA. It is about 75 m above sea level with a maximum depth of 6.1m. The lake's surface area is about 38.5 ha in a sparsely developed suburban area in Sterling.
Access & import/export	Stickleback were collected under annual Fish Resource Permits from the Alaska Department of Fish and Game to MAB as well as the Institutional Animal Care and Use Committee (IACUC 1446584) at Stony Brook University to KRV and MAB and from The College of New Jersey Institutional Care and Use of Animals Committee (protocols 1908-001MW1A1 and 2002-001MW1A3) to MW.
Disturbance	On each sampling event we set up minnow traps. If five repeated sampling events fail to catch our target sample size we will cease further collection efforts for that month. Because tickleback are very abundant and swim readily into minnow traps, we can collect our samples within 1-3 sampling sets at a single effort and therefore pose minimal disturbance to the lake ecosystem. Some non-target species could be caught in minnow traps. All non-target species are immediately released without harm.

Reporting for specific materials, systems and methods

We require information from authors about some types of materials, experimental systems and methods used in many studies. Here, indicate whether each material, system or method listed is relevant to your study. If you are not sure if a list item applies to your research, read the appropriate section before selecting a response.

Materials & experimental systems

n/a	Involved in the study	
☒	☐	Antibodies
☒	☐	Eukaryotic cell lines
☒	☐	Palaeontology and archaeology
☐	☒	Animals and other organisms
☒	☐	Clinical data
☒	☐	Dual use research of concern
☒	☐	Plants

Methods

n/a	Involved in the study	
☒	☐	ChIP-seq
☒	☐	Flow cytometry
☒	☐	MRI-based neuroimaging

Animals and other research organisms

Policy information about studies involving animals; ARRIVE guidelines recommended for reporting animal research, and Sex and Gender in Research

Laboratory animals	Not applicable
Wild animals	All non-target species that are caught in our traps are immediately released. In addition, when traps catch more fish than our IACUC

Wild animals	allowed, we immediately release the rest back into the lake. We clip fins from stickleback fish for DNA extraction and the carcass are stored in 70% ethanol for future research.
Reporting on sex	Not applicable
Field-collected samples	We clip fins from stickleback fish for DNA extraction and the carcass are stored in 70% ethanol for future research.
Ethics oversight	Stickleback were collected under annual Fish Resource Permits from the Alaska Department of Fish and Game to MAB as well as the Institutional Animal Care and Use Committee (IACUC 1446584) at Stony Brook University to KRV and MAB and from The College of New Jersey Institutional Care and Use of Animals Committee (protocols 1908-001MW1A1 and 2002-001MW1A3) to MW.

Note that full information on the approval of the study protocol must also be provided in the manuscript.

Plants

Seed stocks	Not applicable
Novel plant genotypes	Not applicable
Authentication	Not applicable
